# Burden of osteoporosis in Vietnam: An analysis of population risk

**DOI:** 10.1371/journal.pone.0252592

**Published:** 2021-06-16

**Authors:** Duy K. Hoang, Minh C. Doan, Linh D. Mai, Thao P. Ho-Le, Lan T. Ho-Pham

**Affiliations:** 1 Bone and Muscle Research Group, Ton Duc Thang University, Ho Chi Minh, Vietnam; 2 Faculty of Applied Sciences, Ton Duc Thang University, Ho Chi Minh, Vietnam; 3 Department of Internal Medicine, Pham Ngoc Thach University of Medicine, Ho Chi Minh, Vietnam; 4 Garvan Institute of Medical Research, Sydney, Australia; 5 Faculty of Engineering and Information Technology, Hatinh University, Hà Tĩnh, Vietnam; Medical College of Wisconsin, UNITED STATES

## Abstract

**Purpose:**

To estimate the proportion of men and women aged 50 years and older who would be classified as "high risk" for fracture and eligible for anti-fracture treatment.

**Methods:**

The study involved 1421 women and 652 men aged 50 years and older, who were recruited from the general population in Ho Chi Minh City, Vietnam. Fracture history was ascertained from each individual. Bone mineral density (BMD) was measured at the lumbar spine and femoral neck by DXA (Hologic Horizon). The diagnosis of osteoporosis was based on the T-scores ≤ -2.50 derived from either femoral neck or lumbar spine BMD. The 10-year risks of major fractureand hip fracture were estimated from FRAX version for Thai population. The criteria for recommended treatment were based on the US National Osteoporosis Foundation (NOF).

**Results:**

The average age of women and men was ~60 yr (SD 7.8). Approximately 11% (n = 152) of women and 14% (n = 92) of men had a prior fracture. The prevalence of osteoporosis was 27% (n = 381; 95% CI, 25 to 29%) in women and 13% (n = 87; 95% CI, 11 to 16%) in men. Only 1% (n = 11) of women and 0.1% (n = 1) of men had 10-year risk of major fracture ≥ 20%. However, 23% (n = 327) of women and 9.5% (n = 62) of men had 10-year risk of hip fracture ≥ 3%. Using the NOF recommended criteria, 49% (n = 702; 95% CI, 47 to 52%) of women and 35% (n = 228; 95% CI, 31 to 39%) of men would be eligible for therapy.

**Conclusion:**

Almost half of women and just over one-third of men aged 50 years and older in Vietnam meet the NOF criteria for osteoporosis treatment. This finding can help develop guidelines for osteoporosis treatment in Vietnam.

## Introduction

Fragility fracture is a consequence of osteoporosis, the condition characterized by low bone mass and deteriorated microarchitecture of the bone. Fracture imposes a significant public health burden, because it is associated with increased risk of mortality and health care cost. Previous studies have suggested that individuals with an existing fracture have a two-fold increase in mortality. The one-year risk of mortality among women with a hip fracture is estimated to be 21% [1] which is the same as the risk of mortality among women with invasive breast cancer. According to an estimate in the United States, the cost associated with osteoporosis was $16 billion in 2002 [2]. Taken together, osteoporosis and its consequence of fracture represent an important public health problem, and the problem is expected to exacerbate in the future as the population is rapidly aging.

One of the critical question of public health significance is how many women and men are at high risk of fracture in the general population. In the absence of a prospective study, one way to estimate the burden of fracture is by using the distribution of bone mineral density (BMD) to estimate the proportion of individuals with osteoporosis and osteopenia according to the World Health Organization’s recommended criteria [3]. Another way is to use a fracture risk assessment model such as FRAX [4] or Garvan [5]. The FRAX model was developed using population-based cohorts of Caucasian and Asian backgrounds. The FRAX model uses 12 risk factors to produce 10-year risk of major fracture and hip fracture, and the risk can be calibrated in a local population. One way to define "high risk" is to use the criteria recommended by the US National Osteoporosis Foundation (NOF). The NOF recommends that postmenopausal women and men aged 50 years and older should be considered for treatment if they have a prior fracture, a T-score of −2.5 or lower at the femoral neck or lumbar spine, or osteopenia and a FRAX 10-year risk of at least 3% for hip fracture or at least 20% for major osteoporotic fracture [6]. Based on these criteria, it has been estimated that 72% of women aged 65+ years would be eligible for drug therapy [7], and this is a significant burden.

Vietnam is a developing country with a population of 97 million. However, until now there is a lack of population-based prospective data concerning fracture incidence, and as a result, the burden of osteoporosis and fracture is not known. Therefore, we conducted this study to estimate the proportion of Vietnamese women and men who would be eligible for drug treatment is lower than that in the US.

## Study design and methods

The present study was designed as a cross-sectional investigation, and it was part of the Vietnam Osteoporosis Project [8]. The Study’s procedure and protocol were approved by the research and ethics committee of the People’s Hospital 115, and written informed consent was obtained from each individual. Participants were recruited from the general population in Ho Chi Minh City in 2015–2016 period. We approached community organizations (e.g., churches, temples, retiree associations) to invite individuals to participate in the study. We further ran a campaign in television and newspapers to talk about the research project, and through which participants joined the study. Individuals agreed to participate in the study were then transported to the Bone and Muscle Research Laboratory at the Ton Duc Thang University for clinical assessment and evaluation. There was no financial incentive involved, but participants were entitled to a free health check-up and lipid analyses.

In this present study, we included individuals aged 50 years and older at study entry (n = 2093). However, there were 20 participants whose data were not complete for FRAX calculation, and they were further excluded from the study. Ultimately, the present study included 2073 individuals with complete data.

Data concerning anthropometric factors and clinical history were obtained by a questionnaire that was administered to each participant. The filling of the questionnaire was assisted by a research assistant. The following relevant data were collected: current smoking habit, current alcohol use, personal fracture history, family history of osteoporosis, glucocorticosteroid use, and comorbidities such as rheumatoid arthritis. Height and weight were measured by an electronic portable, wall-mounted stadiometer (Seca Model 769; Seca Corp, CA, USA) without shoes, ornaments, hats or heavy layers of clothing. Body mass index (BMI) was derived as the weight in kilograms divided by the square of the height in meters (kg/m^2^), and categorized into 4 groups: underweight (< 18.5); normal (18.5 to <23.0); overweight (23.0 to <27.5) and obese (≥ 27.5) [9].

Bone mineral density (BMD) at the lumbar spine and femoral neck was measured by a Hologic Horizon DXA (Hologic Corp., Bedford, MA, USA) [10]. For the lumbar spine, we measured BMD from L2 to L4. The densitometer was standardized before each measurement with a phantom. The measurement was conducted by a qualified radiology technologist. Based on 20 individuals, the coefficient of variation in BMD at our lab was 1.5% for the lumbar spine and 1.7% for the hip. T-scores for the lumbar spine and femoral neck were determined for each individual using the previously published reference ranges [11].

We inputted the femoral neck T-score data and clinical data into the FRAX model for Thai population to obtain the 10-year risk of major fracture and 10-year risk of hip fracture. This was done online for each individual in the study. A random 5% of the sample individuals was repeated to check for consistency, and we found that the predicted values were 100% agreement. The choice of FRAX—Thailand was based on the consideration that (a) both Vietnam and Thailand are developing countries in South East Asia, although Thailand is economically more advanced than Vietnam; and (b) both populations share common food culture and lifestyle.

Data were mainly analyzed by descriptive statistical methods. Mean and standard deviation of continuous variables (e.g., BMD and 10-year risk) were calculated for each gender. Categorical data were presented as proportion of total. Based on the T-score data, we determined prevalence of osteoporosis for each 10-year age group stratified by gender. We further determined the proportion of individuals who would be eligible for pharmacologic treatment by the recommendation of NOF [6]: (a) those with a low trauma hip or vertebral fracture; (b) those with T-scores ≤−2.5 at the femoral neck, total hip, or lumbar spine; (c) postmenopausal women and men aged 50 years and older with T-score between -1.0 and -2.5 at the femoral neck, total hip, or lumbar spine and a 10-year risk of major fracture≥ 20%, or 10-year risk of hip fracture ≥ 3%. All analyses were conducted using the R statistical environment [12].

## Results

The study involved 1421 women and 652 men, all aged 50 years and older (**Table 1**). The average (SD) age was ~60 (7.8). The average BMI was 23 kg/m^2^ and there was no significant difference between genders. Using the criteria of BMI ≥ 30 kg/m^2^, approximately 3.7% (n = 52) of women and 27% (n = 174) of men were classified as "obese". About 46% (n = 297) of men self-reported to be current smokers, whereas only 1% (n = 15) was reported by women. Rheumatoid arthritis was reported in 51% of women ad 34% of men. Prior fracture was present on 11% of women and 14% of men.

**Table 1:** Basic characteristics of 1421 women and 652 men.

Variable	Women (n = 1421)	Men (n = 652)	P-value
Age	59.7 (7.8)	59.5 (7.8)	0.579
Weight	54.0 (8.6)	61.5 (9.6)	<0.001
Height	152 (5.4)	162 (5.9)	<0.001
BMI	23.4 (3.3)	23.3 (3.2)	0.271
Previous fracture^1^	152 (10.7)	92 (14.1)	0.030
Current smoking^1^	15 (1.1)	297 (45.6)	<0.001
Alcohol use^1^	28 (2.0)	272 (41.7)	<0.001
Glucocorticoids^1^	103 (7.3)	37 (5.7)	0.218
Rheumatoid arthritis^1^	728 (51.2)	221 (33.9)	<0.001
Femoral neck BMD	0.63 (0.12)	0.72 (0.12)	<0.001
Lumbar spine BMD	0.81 (0.14)	0.93 (0.15)	<0.001

**Notes:** Values are shown in mean and standard deviation (in brackets) for continuous variables, and actual frequency and percentage of total for categorical variables^1^. P-values were derived from t-test (in the case of continuous variables) or Chi squared test (in the case of categorical variables).

**Fig 1:**
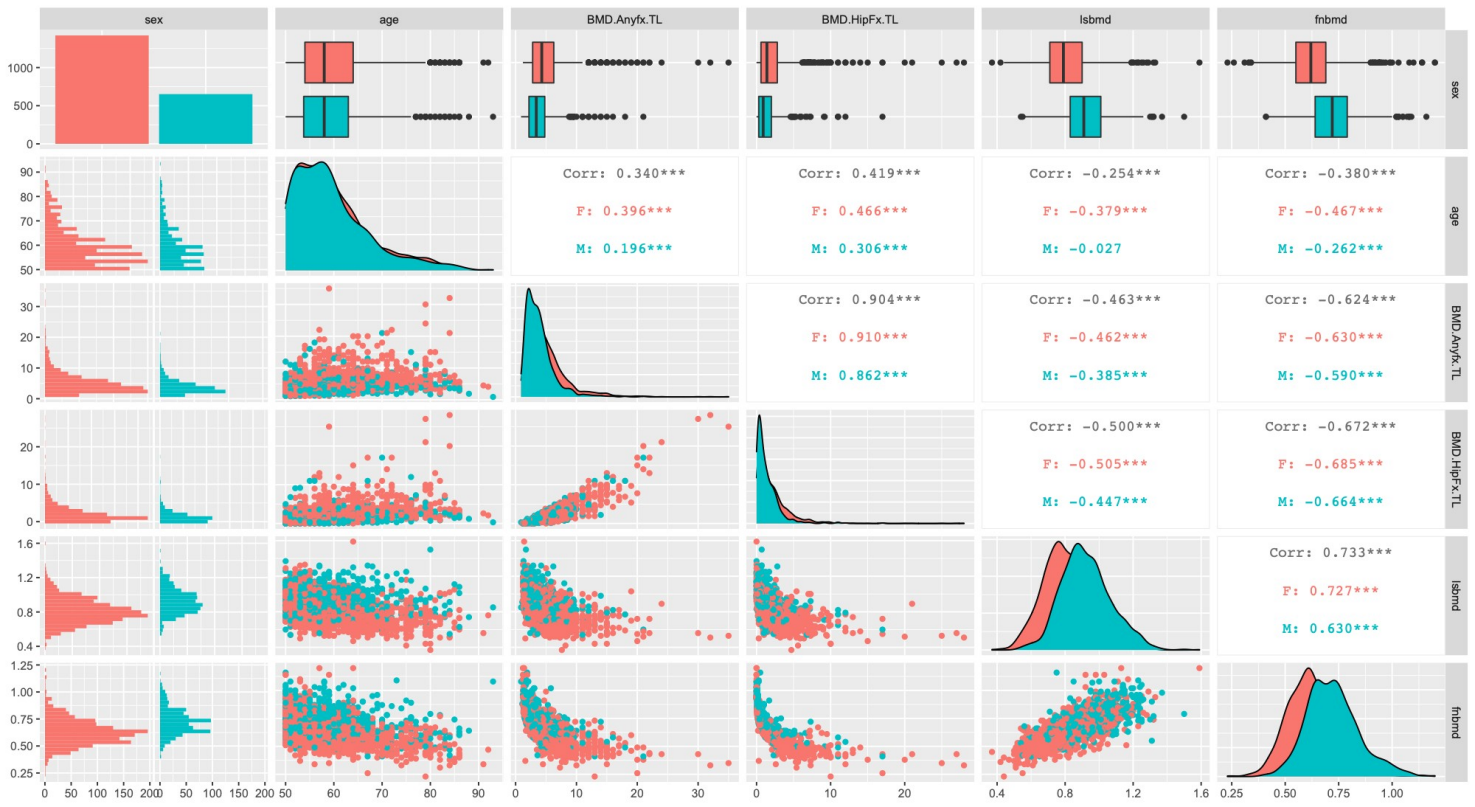
Correlation between age, 10-year risks of fracture ("BMD.Anyfx.TL" and "BMD.Hipfx.TL"), femoral neck BMD ("fnbmd") and lumbar spine BMD ("lsbmd") stratified by gender. The diagonal figures show the histogram of the distribution of each variable. The upper diagonal numbers show the linear correlation coefficient between two variables. The lower diagonal figures visualize the correlation between two variables.

The distribution of 10-year risks of major fracture and hip fracture was skewed toward lower values (**Fig 1**). There was a significant correlation between age and 10-year risk of hip fracture, and the correlation was greater in women (*r* = 0.46) than men (*r* = 0.31). The relationship between 10-year risks of fracture and BMD appears to follow an exponential distribution, with the correlation coefficient ranging from 0.40 to 0.70; however, this should be interpreted with care, because the relationship was not linear.

The distribution of 10-year risks of fracture stratified by age and 10-year age group are shown in **Table 2**. Generally, the 10-year risk was higher in women than men, and in each gender, the risks were increased with advancing age. Approximately 1% (n = 11) of women and 0.1% (n = 1) of men had 10-year risk of major fracture≥ 20%. However, 23% (n = 327) of women and 9.5% (n = 62) of men had 10-year risk of hip fracture ≥ 3%.

**Table 2:** Distribution of FRAX-estimated risk of fracture for men and women.

Gender and risk	Age group			All ages
	50–59	60–69	70+	
**Women**				
10-yr risk of **major** fracture: mean (SD)	4.10 (2.95)	6.04 (3.19)	7.53 (4.53)	5.08 (3.48)
10-yr risk of major fracture≥ 20% (n; %)	2 (0.2)	3 (0.7)	6 (3.5)	11 (0.8)
10-yr risk of hip fracture: mean (SD)	1.39 (1.80)	2.60 (2.12)	4.59 (4.04)	2.13 (2.50)
10-yr risk of hip fracture ≥ 3% (n; %)	98 (11.8)	128 (30.8)	101 (59.1)	327 (23.1)
**Men**				
10-yr risk of major fracture: mean (SD)	3.54 (2.25)	4.33 (2.20)	4.80 (3.01)	3.90 (2.37)
10-yr risk of major fracture≥ 20% (n; %)	0 (0.0)	0 (0.0)	1 (1.4)	1 (0.1)
10-yr risk of hip fracture: mean (SD)	1.06 (1.40)	1.59 (1.39)	2.66 (2.58)	1.39 (1.64)
10-yr risk of hip fracture ≥ 3% (n; %)	25 (6.3)	22 (11.8)	15 (21.4)	62 (9.5)

**Table 3** shows the proportion of individuals that would be eligible for recommended treatment by the NOF criteria. In women, approximately 27% (n = 381) were classified as having osteoporosis at either lumbar spine or femoral neck BMD. Among women with osteopenia, 17.5% (n = 169) were classified as high risk for hip fracture. Thus, in total, 49.4% (n = 702; 95% CI, 48.8% to 52.0%) of women would be recommended for a drug treatment.

**Table 3:** Estimated number of Vietnamese individuals aged 50+ years who are eligible for treatment by the US National Osteoporosis Foundation Guidelines.

Criteria	Women	Men
Prior fracture	152 (10.7)	92 (14.1)
T-scores < = -2.50		
• Femoral neck BMD (FN)	283 (20.0)	35 (5.4)
• Lumbar spine BMD (LS)	261 (18.4)	70 (10.7)
• FN or LS	381 (26.8)	87 (13.3)
Osteopenia at FN or LS	n = 966	n = 425
• 10-yr risk of hip fracture ≥ 3%	169 (17.5)	49 (11.5)
• 10-yr risk of major fracture≥ 20%	2 (0.20)	0 (0.0)
All combined	702 (49.4; 95%CI, 46.8% - 52.0%)	228 (34.9; 95%CI, 31.3% - 38.6%)

**Notes:** CI, confidence interval; Numbers shown are actual number of persons and percentage of sex-specific total (in brackets).

In men, the prevalence of osteoporosis at either lumbar spine or femoral neck was 13.3% (n = 87). Among men with osteopenia, 11.5% (n = 49) were classified as high risk for hip fracture. Combining with the history of fracture (14%), the proportion of men who would be eligible for drug therapy was 35.0% (n = 228, 95%CI, 31.3% - 38.6%).

## Discussion

Although osteoporosis is recognized as a public health concern in Vietnam, there is a lack of data concerning its magnitude. Using a population-based study, we estimate that about 27% of women and 10% of men aged 50 years and older have osteoporosis. We further show that among those with osteopenia, 18% of women and 12% of men would be classified as high risk for hip fracture. If the NOF is used as a recommendation, then about half of women and one-third of men would be eligible for drug treatment. These findings deserve to be elaborated further.

In this study, we found that almost half of women and one-third of men aged 50 years and older would be eligible for treatment under the NOF criteria. The proportion was mainly determined by osteoporosis, followed by osteopenia and FRAX criteria and prior fracture. This finding is important, because it is the first estimate in an Asian population. The proportion was much lower than those estimates by Donaldson et al [7]. However, the difference was largely driven by age group: the Donaldson et al’ study included women aged 65+ years, which was significantly higher than our study’s.

Our estimate of osteoporosis prevalence was actually based on either femoral neck or lumbar spine BMD. If only femoral neck BMD was considered, the prevalence would be lower: 20% in women and 5% in men. In a previous study in the United States the prevalence of osteoporosis (based on either femoral neck or lumbar spine BMD) was ~15% in women and 4.3% in men [13]. In China, the prevalence of osteoporosis was estimated at 25% in women and 15% in men [14]. In Korea, data from the Korea National Health and Nutrition Examination Survey indicated that the prevalence of osteoporosis in was 38% in women and 7.3% in men aged 50 years and older. It is not apparent why the prevalence estimates were different between populations. However, factors such as sampling techniques, characteristics of participants, and reference ranges contribute to the differences.

We found that the FRAX appears to underestimate the risk of fracture in this population. Using the 10-year risk of major fracture threshold of 20%, only 0.8% of women and 0.1% of men would be classified as "high risk". It has been documented in several studies that FRAX tends to underestimate the risk of fracture [15–18]. However, 10-year risk of hip fracture seems to be more sensitive: 23% of women and 10% of men would be classified as "high risk". These data suggest that for assessment of fracture, the 10-year risk of hip fracture index seems reasonable.

There was a moderate agreement between femoral neck BMD and FRAX-based risk of major fracture. Among women with osteoporosis (by femoral neck T-score), FRAX identified only 4% as high risk; this proportion for men was 3%. However, if FRAX estimates of hip fracture risk were used, the proportion of high risk in osteoporosis group was 86% and 66% in women and men, respectively. These findings underline the that when it comes to fracture risk assessment, FRAX-based risk of hip fracture should be the indicator of use.

Osteoporosis is a public health problem, and our finding has important public health implications. Treatment of women with osteoporosis or a prior fracture reduces their fracture risk [19] and mortality [20]. Recent evidence suggests that treatment of women with osteopenia could also reduce their fracture risk by ~40% and death risk by ~35% [21]. However, treatment uptake has been low, with less than 30% of women and less than 10% of men being received treatment [22,23]. At present, fracture risk assessment has not been formally considered in treatment guidelines in Vietnam, and our data can help develop future guidelines.

In this study, we could not evaluate the sensitivity and specificity of FRAX in fracture risk prediction, because this was a cross-sectional study. Still, the sensitivity and specificity of a risk assessment tool is dependent on the threshold that can be considered "high risk". In this study, the thresholds were 20% for major fracture and 3% for hip fracture, and they were derived from the US population. Whether these thresholds are appropriate for Asian population requires further studies.

The present study had strengths and limitations. A major strength of the study is that it was based on a reasonable large sample size. The measurement of BMD was done by a state of the art technology which ensure the study’s internal validity. However, in this study, we used FRAX for the Thai population to estimate 10-year risks of fracture, and because Thailand is more economically advanced than Vietnam, the risks of fracture could have been biased toward higher values. The participants in this study were from a major city, and the finding may not be generalized to rural populations. Moreover, participants were mainly volunteers, and the study might have selected more healthy people which could bias the study results.

In summary, we found almost half of women and one-third of men aged 50+ years of Vietnamese background would be eligible for treatment by the NOF guidelines. This finding can help inform treatment guidelines in Vietnam.
